# Primary tracheal schwannoma: Bronchoscopic management of a rare tracheal tumour

**DOI:** 10.1002/rcr2.1390

**Published:** 2024-05-27

**Authors:** Krizelle Acibal, Belgundi Preeti Vidyasagar, Harikishan Gonuguntla

**Affiliations:** ^1^ Division of Interventional Pulmonology Yashoda Hospital Secunderabad India; ^2^ Interventional Pulmonologist Yashoda Hospital Secunderabad India; ^3^ Lead Consultant Interventional Pulmonologist Yashoda Hospital Secunderabad India

**Keywords:** benign airway obstruction, blue light imaging (BLI), linked colour imaging (LCI), schwannoma, trachea

## Abstract

Primary tracheal tumours are extremely rare, that originate from Schwann cells. We report a case of a primary tracheal schwannoma. A 60‐year‐old male who presented with noisy breathing, shortness of breath, and blood streaked phlegm. Chest CT scan showed an endotracheal mass which was resected bronchoscopically using Rigid bronchoscopy, electrocautery snare and cryoextraction. Biopsy confirmed the diagnosis of schwannoma.

## Graphical abstract

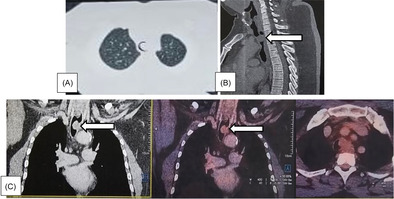

## INTRODUCTION

Primary tracheal tumours are extremely rare and comprise only 1% of all neoplasms. Among these only a quarter are benign.^1^ Benign neoplasms of trachea could be classified according to their cell origins including: neural (carcinoid, benign clear cell, neurofibroma, myoblastoma, and schwannoma), epithelial (papiloma, chondroma, lipoma, fibroma, and fibrous histiocytoma), and mesenchymal (hamartoma). Benign neurogenic tumours are extremely rare and present at any age. This type of tumour arises from nerves located inside the tracheal wall.^2^ Tracheal schwannomas are among the rarest of tracheal tumours and there are no definitive guidelines for management. We present a case of primary tracheal schwannoma which was resected using bronchoscopy.

## CASE REPORT

**FIGURE 1:**
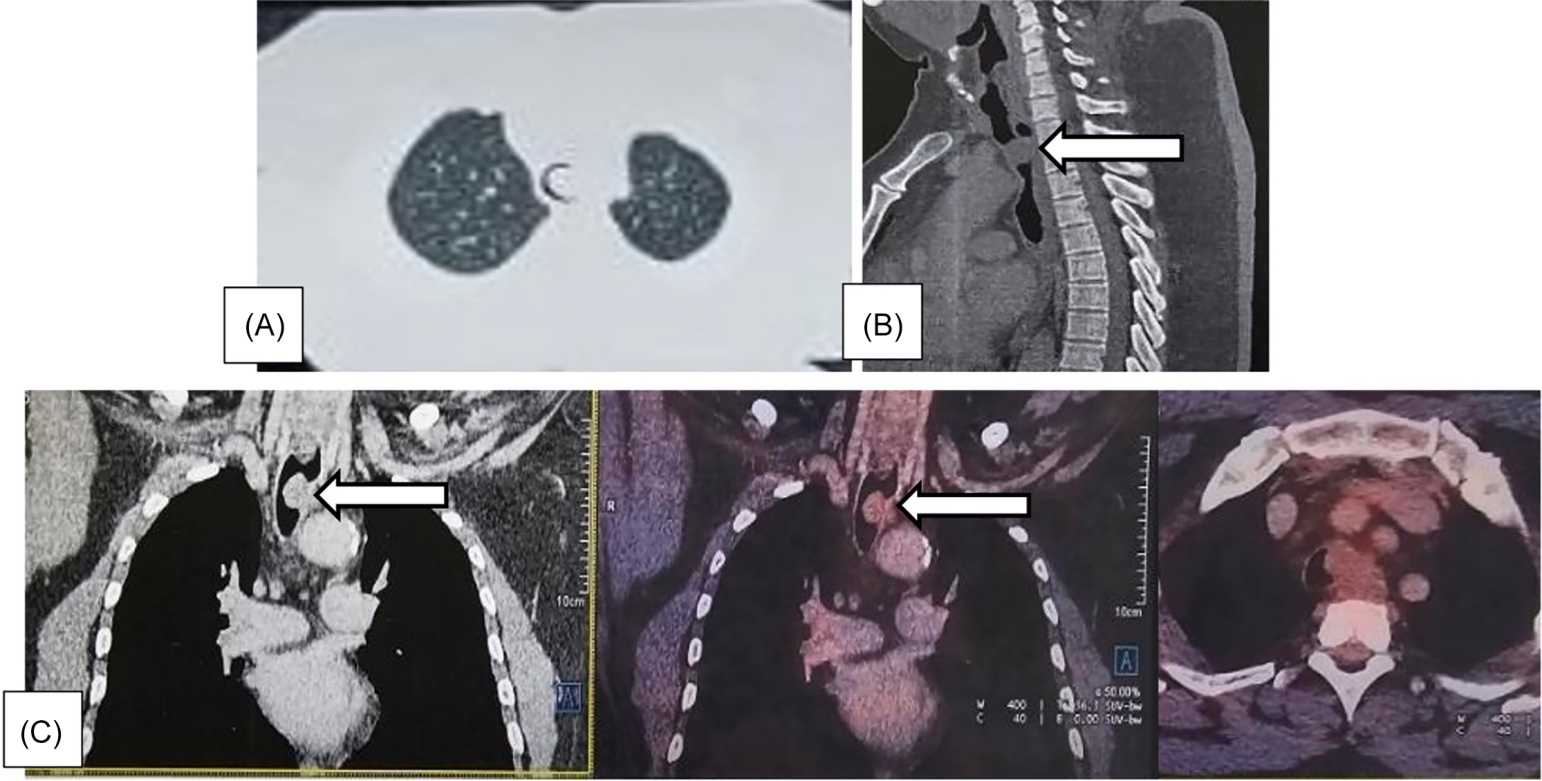
Chest computerized tomography and positron emission tomography‐CT. (A). Axial CT image and (B) sagittal view showing the tumour inside the lumen of the trachea arising from the left lateral wall (arrow). (C) PET‐CT scan image showing low grade FDG avid soft tissue mass with intraluminal component/occlusion noted involving the trachea at the level of manubrium sternum (arrow).

A 60‐year‐old male, never smoker, without comorbid illness, initially presented with a 6 months history of noisy breathing, shortness of breath and blood‐streaked phlegm. Evaluation in a primary hospital revealed a lobulated tracheal mass on chest CT scan with contrast (Figure 1A,B). Pertinent physical examination revealed a Body Mass Index of 37.5 kg/m^2^, with stridor upon auscultation. Laboratory test and chest radiography were normal. PET CT scan was done during admission in our institution which revealed low grade FDG avid soft tissue mass with intraluminal component/occlusion noted involving the trachea at the level of manubrium sternum (D1‐D2 thoracic vertebrae) predominantly arising from the left lateral wall, with maximum dimensions measuring 2.2×1.4 cm (SUV max 3.6) without evidence of oesophageal invasion (Figure 1C).

**FIGURE 2:**
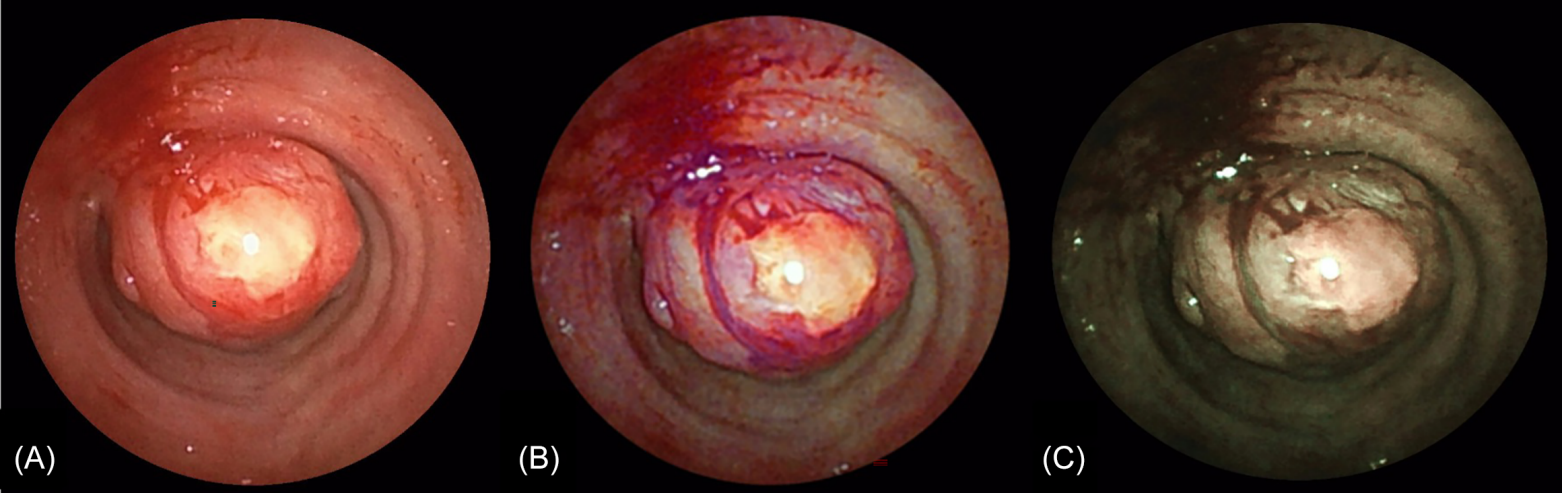
Bronchoscopy image using (A) white light Image (WLI) and the new advanced bronchoscopic optical technology, (B) linked colour image (LCI) and (C) Blue light Image (BLI). The submucosal vascularity and tumour margin on the bronchial mucosa were better visible on LCI than on WLI.

**FIGURE 3:**
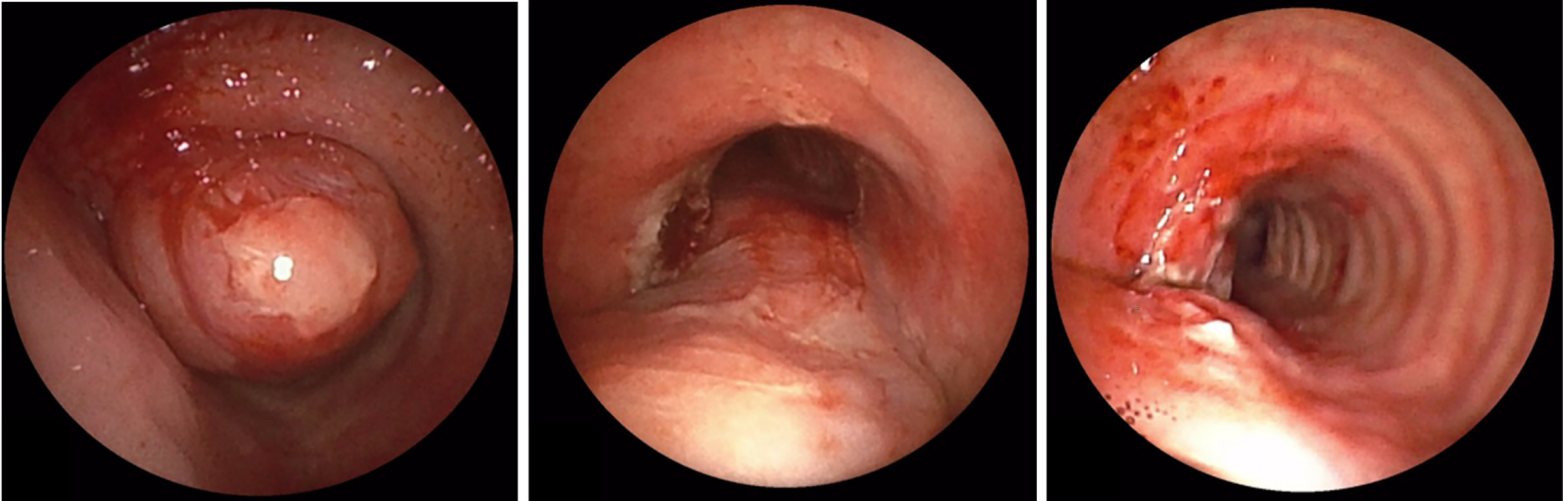
Bronchoscopy images (A) showing a polypoid tumour with a smooth surface with near total obstruction of the tracheal lumen, (B) immediate post‐intervention bronchoscopic findings with complete removal of the mass and (C) 1 week after bronchoscopic intervention.

Flexible bronchoscopic examination while on general anaesthesia revealed a smooth, lobulated mass obstructing more than 80% of the airway lumen in the proximal trachea (Figure 2A and 3A). Cryobiopsy using ERBE cryoprobe 1.1 was sent for frozen section which showed benign mesenchymal lesion. A microlaryngeal (MLS) tube size 5 was passed beyond the tumour to maintain ventilation during the procedure. Using a FUJIFILM EB710P bronchoscope, abnormalities on the lesion were analysed utilizing the Blue light Imaging (BLI) mode and Linked Colour Imaging (LCI) mode (Figure 2). The tumour margin and the vascularity of lesion were best visualized using BLI and LCI as compared to WLI. Bronchoscopic resection was performed by passing on the Rigid bronchoscopy (Efer Dumon size 11) on the side of the MLS tube. Snare electrocautery was used with the blunt coagulation probe to debulk the lesion completely. A cryobprobe 1.1 was used to extract the tumour pieces. Following bronchoscopic resection, total luminal patency was obtained (Figure 3B), and the patient was extubated on the operating table. Procedure was uneventful. The patient was kept on bronchoscopic surveillance.

**FIGURE 4:**
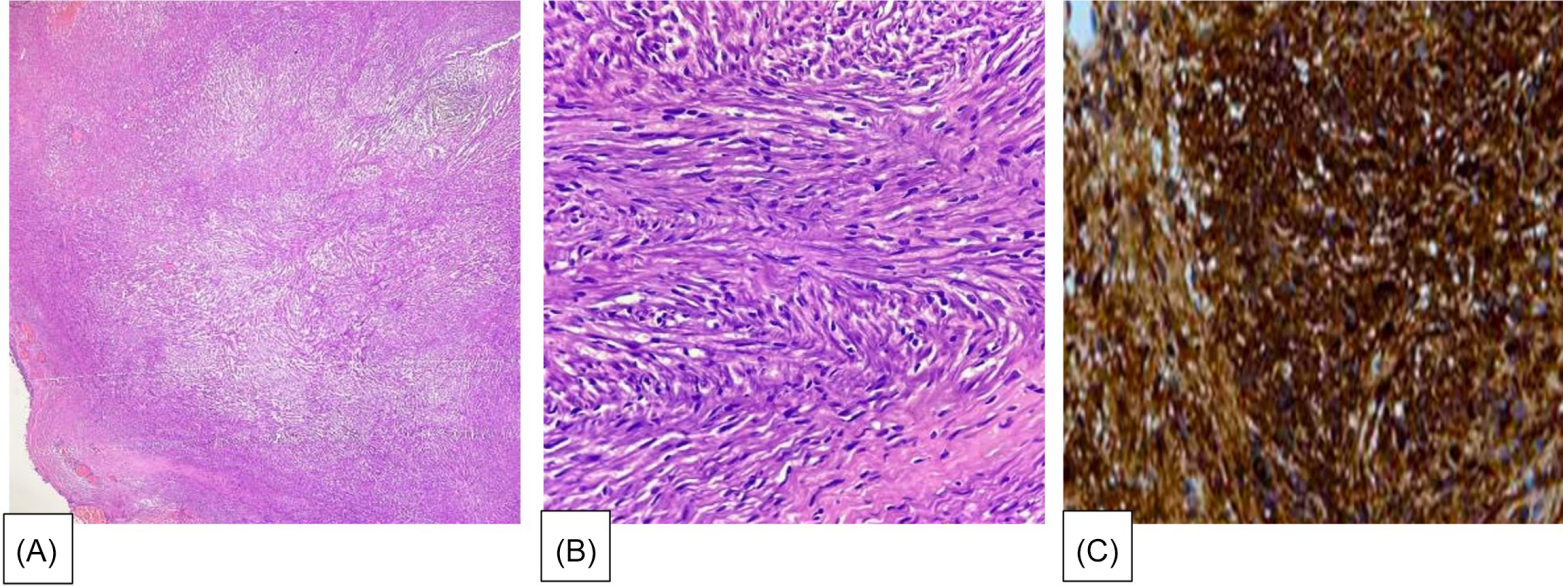
Microscopic images using hematoxilyn and eosin (H&E) stain, on (A) low power objective (LPO) and (B) High power objective (HPO), showing lesion is lined by respiratory epithelium which is composed of spindle cells in short and long fascicles. (C) Immunohistochemistry (IHC) study showed immunoreactivity for S‐100 protein.

Histopathological examination showed fragmented bits of lesion lined by respiratory epithelium which is composed of spindle cells in short and long fascicles. The cells show spindly nuclei without atypia and eosinophilic cytoplasm. Negative for atypia, mitosis, and necrosis. Immunohistochemical staining revealed diffuse positive for S100 and SOX‐10, negative for CK, SMA and Mib, compatible with a diagnosis of tracheal schwannoma (Figure 4).

## DISCUSSION

Neurogenic tumours are divided into nerve sheath tumours and neurofibromas based on their histo‐pathologic features and the existence of nerve axon. Nerve sheath tumours have no axon, and are categorized as schwannomas (neurilemmoma) and non‐schwannian nerve sheath tumours. Schwannomas of the trachea arise from intraluminal neurogenous tissue, more precisely, schwann cells of the nerve sheath. On the other hand, neurofibromas consist of all elements of nerve including schwann cells, perineural cells and axons.^3^ Schwannoma has shown to be more common among women while neurofibroma has been reported only in men. Majority of the cases have occurred among adults^4^ and most frequently observed in the distal of trachea, followed by decreasing order in the proximal and middle thirds.^2^, ^4^ Verocay et al first described schwannoma, and attributed its histogenesis to the Schwann cells of the neural sheaths.^2^

Signs and symptoms are non‐specific. As with other intraluminal tumours, symptoms of airway obstruction, such as coughing, wheezing, dyspnea, shortness of breath can be present. The tumour must be large enough to obstruct more than 50% of the diameter of the trachea before dyspnea occurs. Less frequent symptoms are hemoptysis, chest pain and hoarseness.^2^, ^4^ The patient presented in this case had cough, breathlessness and hemoptysis.

Preoperative diagnosis is difficult for rare cases like tracheal schwannoma as the signs and symptoms are non‐specific and often varies. Hence, diagnosis is usually delayed. Imaging studies can be used to detect such cases. Chest radiograph is not a sensitive modality to detect schwannoma as there will be superimposed soft tissues and bony structures^3^ but in other cases it could demonstrate the tracheal mass and outline its intraluminal extension and secondary complications such as collapse due to pneumonia.^4^ A multislice computerized tomography with contrast is more sensitive and used to delineate tumour size, site and extratracheal extension.^3^, ^5^ PET/CT can also be done to stratify the risk of the intraparenchymal nodules if present, rather than to investigate the tracheal lesion. The elevation of FDG uptake in schwannomas is known and does not predict malignancy.^5^ As mentioned in the case, our patient underwent chest radiograph, but there was no suspicious findings of endotracheal lesion, while Chest CT scan and PET/CT scan revealed the presence of the endotracheal mass.

The definitive diagnosis for tracheal schwannoma is a tissue biopsy through a bronchoscopic technique. It can be done with a flexible or rigid tube and should be done under local or general anaesthesia. Schwannomas are well circumscribed and encapsulated tumours. Microscopically, they have regions of high and low cellularity, called Antoni A and B areas, respectively.^3^, ^6^ In highly differentiated Antoni A areas, there may be nuclear palisading, whorling of the cells and Verocay bodies. Immunohistochemical stains for S‐100 protein and Sox10 are positive.^3^, ^6^, ^7^ The same findings were also observed in our case.

The new bronchoscopic optical technology, such as Linked Colour Imaging (LCI) and Blue Light Imaging (BLI), may be useful for the evaluation of endoluminal benign and malignant airway lesions. Linked colour imaging (LCI) and Blue Light imaging (BLI) are novel technologies developed by Fujifilm Corporation. LCI and BLI use narrowband short wavelength light. BLI uses blue and green colour information to produce red colour‐enhanced images. LCI uses the information of all three colours. Unlike conventional white light imaging (WLI), the captured image is output with colour enhancement in its own colour range (e. g., red is changed to vivid red and white to clear white) by unique image processing.^8^ This combination of light provides more information about the vasculature and architecture of the mucosal surface than that obtained with conventional white light imaging (WLI). BLI has higher emission intensity at short wavelengths and is more suitable in the diagnosis of intrapapillary capillary loops in such areas than LCI.^9^ However, LCI findings do not reflect the pathological differences because they show just submucosal colour changes.^10^ As mentioned in the case, we have used all novel image‐enhancement technology mode to further evaluate the mass.

The rarity of these lesions has led surgeons to practice different surgical techniques but the primary treatment is surgical excision. No standard procedure has been adopted yet. Endoscopic excision, sleeve excision or tracheal resection, are all commonly accepted treatment modalities.^7^ The choice of treatment should be influenced by the clinical presentation, whether the tumour is pedunculated or sessile, and the presence or absence of an extra‐tracheal component. Endoscopic treatment, including laser, electrocautery snaring, argon plasma coagulation, cryotherapy, endoscopic excision and microdebridement, is probably appropriate if the tumour is pedunculated and has no demonstrable extratracheal component, on the otherhand, tracheal resection and primary anastomosis is advised for more involved tumours.^2^ The duration of follow up after surgical intervention has not been consistent throughout the literature, mainly because of the rarity of the lesion. In our case, we utilized bronchoscopic approach using electrocautery snare and cryotherapy.

In conclusion, primary tracheal schwannoma is an extremely rare benign tumour that can occur with non‐specific signs and symptoms. Chest CT scan and bronchoscopic evaluation are useful for the diagnosis. The definitive treatment depends on the condition of the patient as well as the bronchoscopic appearance of the mass. Although the prognosis is favourable with low recurrence rate, long term imaging follow up and bronchoscopic surveillance of tumour recurrence is required.

## AUTHOR CONTRIBUTIONS

The authors indicated in parenthesis made substantial contributions to the following tasks: *Conceptualization*: H.K.G. *data collection*: K.L.A. *Draft manuscript preparation*: K.L.A. and P.V. All Authors reviewed the paper and approved the final version of the manuscript.

## CONFLICT OF INTEREST STATEMENT

None declared.

## ETHICS STATEMENT

The authors declare that appropriate written informed consent was obtained for the publication of this manuscript and accompanying images.

## Data Availability

The data that support the findings of this study are available on request from the corresponding author. The data are not publicly available due to privacy or ethical restrictions.
